# Early intervention in psychosis services: A systematic review and narrative synthesis of barriers and facilitators to seeking access

**DOI:** 10.1192/j.eurpsy.2023.2465

**Published:** 2023-11-06

**Authors:** Jacqui Tiller, Tess Maguire, Katherine Newman-Taylor

**Affiliations:** ^1^School of Psychology, University of Southampton, Southampton, UK; ^2^Psychology Department, Southern Health NHS Foundation Trust, Southampton, UK

**Keywords:** barriers, duration of untreated psychosis, early intervention, facilitators, psychosis, qualitative synthesis, systematic review

## Abstract

**Background:**

The duration of untreated psychosis (DUP) continues to be a global priority. Early intervention services were established to reduce treatment delays but have had limited impact. This systematic review examines barriers and facilitators to seeking access to these services, to identify targets for service level change.

**Methods:**

We conducted a systematic review of relevant databases (PsychINFO, MEDLINE, CINAHL, and PsychARTICLES) using pre-defined search terms for *psychosis, early intervention,* and *barriers and facilitators.* Given the majority of qualitative studies, a thematic synthesis rather than meta-analysis was indicated.

**Results:**

The search yielded 10 studies. Mental health stigma and discrimination predict DUP, compounded by structural barriers which limit the impact of early intervention services on timely access to recommended treatments. Synthesis of the qualitative studies generated three themes: *knowledge, relationships*, and *stigma.* Lack of knowledge, absence of supportive relationships (social and professional), and self-stigma constitute significant barriers to seeking access to early intervention services.

**Conclusions:**

This is the first review of the barriers and facilitators to seeking access to early intervention services. The findings highlight public health and secondary care service targets to expedite access to recommended treatments and thereby reduce the DUP.

## Introduction

*Duration of untreated psychosis* (DUP) describes the period between initial psychotic symptoms and engagement in recommended treatments, and typically lasts 1–2 years [1, 2]. Delayed access to treatment predicts poorer clinical and social outcomes up to 8 years later [3–6]. This comes at considerable personal and healthcare costs [7–9], leading the World Health Organization [10] to identify DUP as an international healthcare target.

Specialist early intervention services have been established in Australia, New Zealand, and the UK, and more recently in North America, Asia, Scandinavia, and other European countries, with the aim of identifying and treating early symptoms of psychosis over the initial *critical period* [11–13]. These services have been well received by young people with psychosis [14], with some evidence of improved outcomes [15]. Disappointingly, however, the expectation that this step change in service delivery would lead to overall reductions in DUP is not (yet) supported by the literature [16], leading to calls to identify and target barriers and facilitators to accessing these services [17].

A recent systematic review of the barriers and facilitators to successful *implementation* of early intervention services highlighted systemic (e.g., funding and organizational structures), service (e.g., coherence of provision), and staff (e.g., knowledge and attitudes) factors [18]. A linked but distinct question concerns the factors affecting the likelihood that people will *seek access* to early intervention services. To our knowledge, this is the first review of barriers and facilitators to seeking access to early intervention for psychosis services.^1^

## Methods

Broad methodological alignment with O’Connell et al. [18] allows comparison across these two complementary reviews.

### Pre-registration and search procedure

The review was pre-registered on PROSPERO (ID: CRD42022377155) and follows the preferred reporting guidelines for systematic reviews (PRISMA) [21]. We searched four electronic databases on 18.09.23 (PsychINFO, MEDLINE, CINAHL, and PsychARTICLES) using free text and subject headings (where applicable) to improve search accuracy (see Table 1). Additionally, we searched ProQuest, Ethos, and British Library databases for gray literature to ensure a comprehensive search and reduce the risk of publication bias [22].

**Table 1.** Free text and subject headings

	Psychosis	Early intervention	Barriers and facilitators
Free text	Schizo* OR Psychotic* OR Psychosis* OR “Schizophren* spectrum*” OR “Acute psychosis”	“Early onset” OR “First onset” OR “First episode”	Barrier* OR Challenge* OR Obstacle* OR Access* OR Facilita* OR Enabl* OR Disengag* OR Engag*
PsychINFO subject headings	“Schizophrenia” OR “Psychosis”	“Early intervention” OR “First episode (disorders)”	No relevant terms are available
MEDLINE subject headings	MM “Schizophrenia” OR MM “Psychotic disorders”	No relevant terms are available	No relevant terms are available
CINAHL subject headings	“Schizophrenia” OR “Psychotic disorders”	“Early intervention”	No relevant terms are available
PsychARTICLES subject headings	“Schizophrenia” OR “Psychosis”	“Early intervention” OR “First episode (Disorders)”	No relevant terms are available

### Inclusion and exclusion criteria

Table 2 outlines study eligibility criteria, following Butler et al. [23]. The search was not limited by publication date or status, to ensure a balanced summary of the evidence and reduce the impact of publication bias [24].

**Table 2.** Inclusion and exclusion criteria

	Inclusion	Exclusion
Population	Participants ≥14 years old Psychosis or psychosis-type experiences (e.g., schizophrenia, schizoaffective disorder, first episode psychosis, drug-induced psychosis)	Participants <14 years old Participants identified as experiencing: at-risk mental states prodromal experiences of psychosis prolonged psychosis comorbid mental health condition in addition to psychosis Carers or family members as participants Staff as participants Service provider views/accounts
Phenomena of interest	Views/perspectives on barriers and/or facilitators to accessing early intervention for psychosis services	No consideration of individuals’ experiences relevant to initial access to early intervention services (e.g., help-seeking once contact is made)
Context	Early intervention for psychosis services Early access to care for psychosis	Community mental health teams Inpatient treatment settings
Study design	Quantitative or qualitative research – published or unpublished	Books, chapters, book reviews, commentaries
Other	Available in English	Not written in English

The perspectives of carers, family members, and staff are also important in understanding access to services. However, these may diverge in important ways from the views of service users themselves, and so we focus on people with psychosis in the current review.

### Study selection, data extraction, and analysis plan

**Figure 1.**
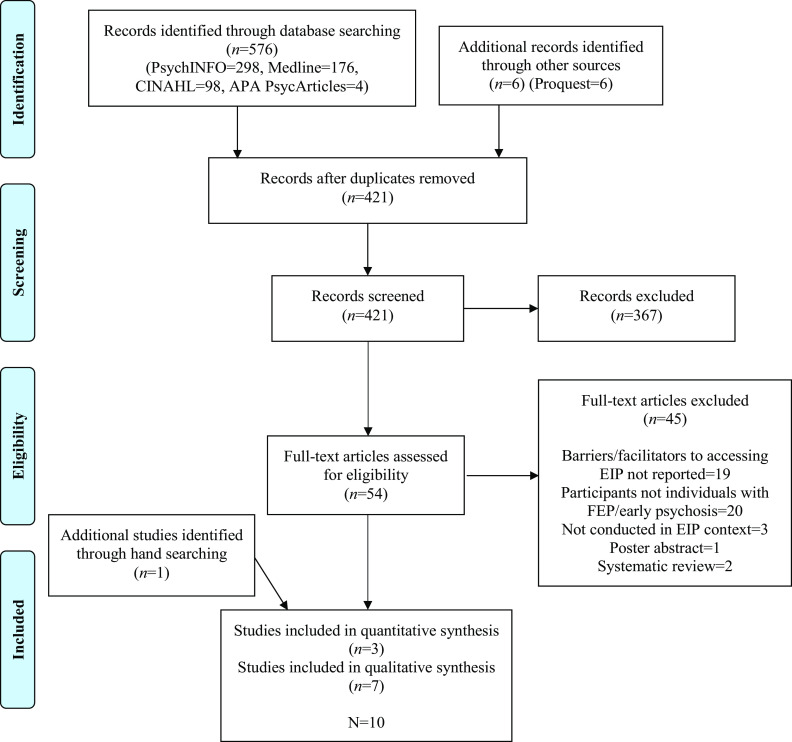
PRISMA diagram for paper selection.

We used Rayyan reference management software [25] to collate search results. The search yielded 582 articles, 421 after duplicates were removed. An independent reviewer second rated 10% of abstracts (*n* = 38) with good agreement (84.2%)^2^ [26]. Full-text screening and hand searching of selected papers resulted in the identification of 10 papers which described three quantitative [17, 27, 28] and seven qualitative studies [29–35] (see Figure 1).

With just three quantitative studies measuring differing primary outcomes, a narrative summary of the characteristics and key results was indicated rather than a meta-analysis. In line with Cochrane recommendations for synthesizing qualitative research, we undertook a thematic synthesis of the qualitative studies [36–38]. This approach is positioned between integrative and interpretative approaches and includes: (1) *line by line coding* of individual study results (for which we used NVIVO, [39]), (2) generating *descriptive themes*, and then (3) generating *analytical themes* which interpret qualitative data across primary studies^3^ [40].

### Quality assessment and risk of bias

The Study Quality Assessment Tool (SQAT) [41] for observational studies, and the Critical Appraisal Skills Programme (CASP) [42] checklist for qualitative studies include 14 and 10 items respectively to assess methodological, analysis, and interpretation bias. In line with previous reviews, we totaled the number of “Yes” responses [cf. 43]. Quantitative studies scored 7/10 relevant domains (see Table 3) and qualitative studies scored at least 7/10 (see Table 4). The key limitation of the quantitative studies was the reliance on cross-sectional data which precludes causal inferences. Though strong in most domains, the majority of qualitative studies failed to address researcher reflexivity and the impact of researcher/participant interactions, which are key to rigorous qualitative designs [44, 45].

**Table 3.** Quality assessment – quantitative studies

First author	Was the research question or objective in this paper clearly stated?	Was the study population clearly specified and defined?	Was the participation rate of eligible persons at least 50%?	Were all the subjects selected or recruited from the same or similar populations (including the same time period)? Were inclusion and exclusion criteria for being in the study prespecified and applied uniformly to all participants?	Was a sample size justification, power description, or variance and effect estimates provided?	For the analyses in this paper, were the exposure(s) of interest measured prior to the outcome(s) being measured?	Was the timeframe sufficient so that one could reasonably expect to see an association between exposure and outcome if it existed?	For exposures that can vary in amount or level, did the study examine different levels of the exposure as related to the outcome (e.g., categories of exposure, or exposure measured as continuous variable)?	Were the exposure measures (independent variables) clearly defined, valid, reliable, and implemented consistently across all study participants?	Was the exposure(s) assessed more than once over time?	Were the outcome measures (dependent variables) clearly defined, valid, reliable, and implemented consistently across all study participants?	Were the outcome assessors blinded to the exposure status of participants?	Was loss to follow-up after baseline 20% or less?	Were key potential confounding variables measured and adjusted statistically for their impact on the relationship between exposure(s) and outcome(s)?	Total (/number of relevant domains)
Archie	Y	Y	Y	Y	N	N	N	NA	Y	NA	Y	NA	NA	Y	7/10
Birchwood	Y	Y	Y	Y	N	N	N	NA	Y	NA	Y	NA	NA	Y	7/10
Kular	Y	Y	Y	Y	N	N	N	NA	Y	NA	Y	NA	NA	Y	7/10

Abbreviations: CD, cannot determine; N, no; NA, not applicable; NR, not reported; Y, yes.

**Table 4.** Quality assessment – qualitative studies

First author	Was there a clear statement of the aims of research?	Is a qualitative methodology appropriate?	Was the research design appropriate to address the aims of the research?	Was the recruitment strategy appropriate to the aims of the research?	Was the data collection in a way that addressed the research issue?	Has the relationship between researcher and participants been adequately considered?	Have ethical issues been taken into consideration?	Was the data analysis sufficiently rigorous?	Is there a clear statement of findings?	How valuable is the research?	Total
Bay	Y	Y	Y	Y	Y	CT	Y	Y	Y	Y	9
Cowan	Y	Y	Y	Y	Y	Y	Y	Y	Y	Y	10
Harris	Y	Y	Y	Y	Y	Y	Y	Y	Y	Y	10
Islam	Y	Y	Y	Y	Y	CT	CT	Y	Y	Y	8
Jansen	Y	Y	Y	Y	Y	CT	Y	Y	Y	Y	9
Jansen	Y	Y	Y	Y	Y	CT	Y	Y	Y	Y	9
Lee	Y	Y	Y	Y	Y	CT	N	CT	Y	Y	7

Abbreviations: CT, cannot tell; N, no; Y, yes.

Quality assessments were completed by two raters independently with excellent agreement (100% SQAT; 95.71% CASP). Initial discrepancies with the CASP were resolved through discussion with the supervisory team. The quality assessment was not used to exclude studies (following Noyes et al. [46] who note that domains are not equally weighted and so cut-off scores are arbitrary).

### Researcher reflexivity

Reflexivity is a key element of qualitative research and requires researchers to consider their own role in the study and how this may influence findings [45]. This study was completed as part of the first author’s doctoral research. The second and third authors are experienced clinicians and researchers in the field. All three are healthcare professionals with experience in collecting data in early intervention services. We reflected on our roles, experiences, and assumptions during the thematic synthesis process to reduce the risk of bias [47].

## Results^4^

### Study characteristics

All three quantitative studies and six of the seven qualitative studies were published, with one unpublished qualitative thesis. All were conducted in the northern hemisphere, though one explored experiences of international students studying abroad and receiving support for first-episode psychosis [35]. The quantitative studies recruited 78–200 majority male participants to observational cohort designs. The qualitative studies recruited 5–24 participants, with a broadly even male: female reported gender mix (though Cowan et al. [30] recruited more men). The majority utilized semi-structured interviews (*n* = 5) and thematic analyses (*n* = 4).

### Key findings

The three quantitative studies examined care pathways to early intervention services to determine barriers to access, the role of stigma specifically, and potential differences with ethnicity (see Tables 5 and 6). Mental health stigma was identified a key barrier to seeking access to services and predicted DUP [28]. Structural barriers within broader mental health services then delayed access to early intervention teams, thereby limiting the impact of these services on reducing DUP [17]. Perhaps unexpectedly, there were no differences in DUP or who initiated help-seeking (the person themselves, family/friends, or police) between ethnic groups, though Asian and other minoritized ethnic groups were more likely than White (×4) and Black (×3) participants to access early intervention via emergency services [27].

**Table 5.** Study characteristics

Authors Country	Title	Aims	Participants age range (*Mean*) gender	Data collection method	Design and analyses
Quantitative studies					
Archie et al. [27] Canada	Ethnic diversity and pathways to care for a first episode of psychosis in Ontario	To investigate pathways to care for different ethnic groups accessing early intervention services in Ontario	*N* = 200 16–50 (White *M* = 24.1; Black *M* = 24.2; Asian *M* = 26.8; Other *M* = 22.6) 78% males		Cross-sectional (secondary analysis) Chi-square; regression
Birchwood et al. [17] UK	Reducing the duration of untreated psychosis: care pathways to early intervention in psychosis services	To identify components in pathways to care during untreated psychosis and contribution to delays in accessing early intervention services To model the impact of targeted changes in care pathways to reduce DUP	*N* = 348^a^ 14–35 (*M* = 21.6 at illness onset) 73% males	Questionnaires	Cross-sectional ANOVA; sensitivity analysis
Kular et al. [28] UK	Stigma and access to care in first-episode psychosis	To investigate associations between mental health stigma and access to care for people with first-episode psychosis	*N* = 89^b^ 14–37 (*M* = 23.2) 72% males	Questionnaires	Cross-sectional Regression
Qualitative studies					
Bay et al. [29] Norway	Obstacles to care in first-episode psychosis patients with a long duration of untreated psychosis	To investigate factors preventing or delaying people with long DUP from accessing services	*N* = 8 17–44 (*M* = not stated) 4 males, 4 females	Semi-structured interviews	Interpretative phenomenological approach
Cowan et al. [30] Canada	Engagement in specialized early intervention services for psychosis as an interplay between personal agency and critical structures: A qualitative study	To investigate factors influencing people’s choices to access, remain involved with, and leave early intervention services	*N* = 24 17–34 (*M* = 22.67) 16 males, 6 females, 2 transgender	Semi-structured interviews	Thematic analysis
Harris [31] UK	Exploring young people’s constructions of a first episode of psychosis	To investigate culture narratives held by young people regarding access to and impact of early intervention services	*N =* 5 22–35 (*M* = 28) 2 males, 3 females	Semi-structured interviews	Narrative analysis
Islam et al. [32] UK	Black and minority ethnic groups’ perception and experience of early intervention in psychosis services in the United Kingdom	To investigate barriers to early intervention services for Black and minority ethnic groups, linked to cultural appropriateness, accessibility, and acceptability	*N =* 22 18–35 (*M* = 22) 11 males, 11 females	Focus groups	Thematic analysis
Jansen et al. [33] Denmark	Service user perspectives on the experience of illness and pathway to care in first-episode psychosis: A qualitative study within the TOP project	To investigate perspectives on helpful and unhelpful pathways to care for people with first-episode psychosis, and barriers to early detection and treatment	*N =* 11 15–24 (*Median* = 20) 6 males, 5 females	Semi-structured interviews	Thematic analysis
Jansen et al. [34] Denmark	Important first encounter: Service user experience of pathways to care and early detection in first-episode psychosis	To investigate people’s experiences of early detection and transition to psychosis services, including pathways to care, illness understanding, and barriers to adequate care	*N =* 10 18–27 (*Median* = 21) 5 males, 5 females	Semi-structured interviews	Thematic analysis
Lee et al. [35] Canada	Challenges in and recommendations for working with international students with first-episode psychosis: Descriptive case series	To identify and describe challenges for international students with first-episode psychosis accessing early intervention services	*N =* 7 14–35 (*M* = not stated) 4 males, 3 females	Chart reviews	Descriptive case series

a Five participants were excluded from analyses due to insufficient information to calculate DUP.

b *N* denotes a subset of the 132 participants in a wider study; demographic details describe the full sample.

**Table 6.** Key findings of the original studies

Authors	Participant and service characteristics	Key findings reported	Limitations
Quantitative studies			
Archie et al. [27]	Schizophrenia spectrum conditions Early intervention services	No differences between ethnic groups for DUP (median = 22 weeks) or initiation of help-seeking by family/friends (53%), self (33%), or police (15%) More similarities than differences in pathways to care across ethnic groups Asian and other minority ethnic groups more likely than White (×4) or Black (×3) participants to use emergency services as first point of contact	Cross-sectional design Retrospective reporting of DUP Inter-rater reliability not established Exclusion of non-English speaking participants
Birchwood et al. [17]	Schizophrenia spectrum conditions Early intervention service	Delays in accessing early intervention services strongly correlated with DUP DUP *prolonged* once people entered mental health services Structural barriers likely to negatively affect impact of early intervention services on reducing DUP	Cross-sectional design Retrospective reporting of DUP 30% of original sample not consented
Kular et al. [28]	First episode psychosis Early intervention services	Associations between total stigma and DUP (*r* = 0.276, *p <* 0.01), discrimination and DUP (*r* = 0.272, *p* = 0.01), and disclosure and DUP *(r* = 0.253, *p <* 0.05) General mental health stigma and perceived discrimination are significant predictors of DUP	Cross-sectional design Retrospective reporting of DUP Other facets of stigma not measured
Qualitative studies			
Bay et al. [29]	Schizophrenia spectrum conditions Wider research subsample	Five themes generated: (1) participants’ failure to recognize symptoms of psychosis; (2) difficulties expressing their experiences; (3) concerns about stigma; (4) poor psychosis detection skills among health-care professionals; (5) participants’ lack of awareness or understanding of informational campaigns	Retrospective accounts Ethnicity not reported Negative symptoms may have affected recall for some participants Service user accounts may not reflect others who are not service users
Cowan et al. [30]	First episode psychosis Early intervention service	Three themes generated: (1) fluidity and temporality of engagement and disengagement; (2) engagement as an ongoing negotiation; (3) critical structures and agency As people’s needs changed, they sought to renegotiate service input but this was constrained by service and societal structures	Retrospective accounts Service user accounts may not reflect others who are not service users
Harris [31]	First episode psychosis Early intervention service	Young Black people’s narratives of their unusual experiences changed over time and linked to dominant social discourses Dominant medical discourses may tacitly reinforce stigmatized identities; biopsychosocial explanations more helpful to some	Retrospective accounts Service user accounts may not reflect others who are not service users
Islam et al. [32]	Diagnoses not specified Early intervention service	Five themes generated: (1) help-seeking; (2) culture and beliefs; (3) social stigma and shame; (4) experience of early intervention services; (5) improving BME access and experiences of services Initial help sought from faith/spiritual healers for many with diverse ethnicities Limited collaboration between mental health services and charity/voluntary organizations to meet individuals’ cultural and spiritual needs.	Retrospective accounts Service user accounts may not reflect others who are not service users
Jansen et al. [33]	Schizophrenia spectrum conditions Early intervention service	Four themes generated: (1) support from significant others; (2) use of the internet as a source of information about psychosis and treatment; (3) lack of knowledge of symptoms or normalization of psychotic symptoms, (4) fear of stigmatization and embarrassment following symptom disclosure	Retrospective accounts Service user accounts may not reflect others who are not service users
Jansen et al. [34]	First episode psychosis Early detection service	Five themes generated: (1) stigma and fear of the psychiatric system; (2) impact of traumatic experiences; (3) importance of significant others in finding the right treatment and supporting help-seeking; (4) experience of safety and trust within the early detection team; (5) relief at receiving a diagnosis “Anti-stigmatized space” within early detection team key to accessing support	Retrospective accounts Service user accounts may not reflect others who are not service users First study of early detection service – requires replication
Lee et al. [35]	First episode psychosis Early intervention service	Four themes generated (barriers to access for FEP international students): (1) difficulty maintaining student visa status; (2) limited social and family support; (3) financial and health insurance issues; (4) service disengagement Unique challenges for international students require specific support	Retrospective accounts Service user accounts may not reflect others who are not service users

Thematic analysis of the qualitative data [36–38] yielded three descriptive themes associated with barriers and facilitators to accessing early intervention for psychosis services: *knowledge*, *stigma*, and *relationships* (see Table 6 and Supplementary Material).

*Knowledge* describes individuals’ experiences in which information (or absence of information) known to the person and their support system (including families and mental health professionals) had a critical impact on whether and when they were able to access early intervention services. All studies identified limited knowledge – whether regarding psychosis symptomology, possible trajectories, and treatment options – as a significant barrier to help-seeking. For example, misattribution of symptoms to depression, drug use, or normal experiences of adolescence [33], believing that symptoms did not warrant treatment [29, 31, 33], and being unaware of services available [29, 32], all delayed help-seeking and therefore access to recommended treatments. When people did seek help, this lack of knowledge could be compounded by that of primary care clinicians (e.g., General Practitioners in the UK) who also misattributed symptoms to anxiety or depression [29, 32], and other relevant professionals (e.g., immigration officials for international students) [35].

By contrast, four studies highlighted the impact of accurate information about psychosis and mental health services, for example from ongoing public health campaigns, on facilitating access [29–31, 33], and that actively seeking additional information helped people develop an understanding of their experiences which in turn prompted help-seeking [31, 33].

*Stigma* of mental health problems was identified in all qualitative studies as a key barrier to seeking access to early intervention services. Participants’ stigmatized beliefs about mental illness, and fears about others’ responses, in line with dominant societal discourses, affected the likelihood of disclosure and help-seeking [29–31, 33–35]. Two studies found that specific fears about being returned to hospital stopped people seeking help [31, 34]. Socio-cultural factors affected stigma and therefore help-seeking and access to services. For example, where dominant narratives were highly stigmatizing of mental illness (and psychosis specifically) people were less likely to seek help from early intervention services [e.g., 31, 32].

The third descriptive theme highlights the impact of quality of *relationships* on likelihood of accessing early intervention services. Consistent emotional and practical support to disclose and manage psychotic experiences day-to-day increased access to services across six studies [30–35], and a lack of supportive familial relationships and friendships was identified as a barrier [31, 35]. Similarly, collaborative relationships with interpersonally effective professionals that support autonomy and shared decision-making, and flexible service systems (e.g., regarding the pace of engagement), facilitated help-seeking and maintenance of early engagement with services [30–32; 34]. Given the typical age of onset for psychosis, parental relationships were both a key facilitator and barrier [33, 34].

**Figure 2.**
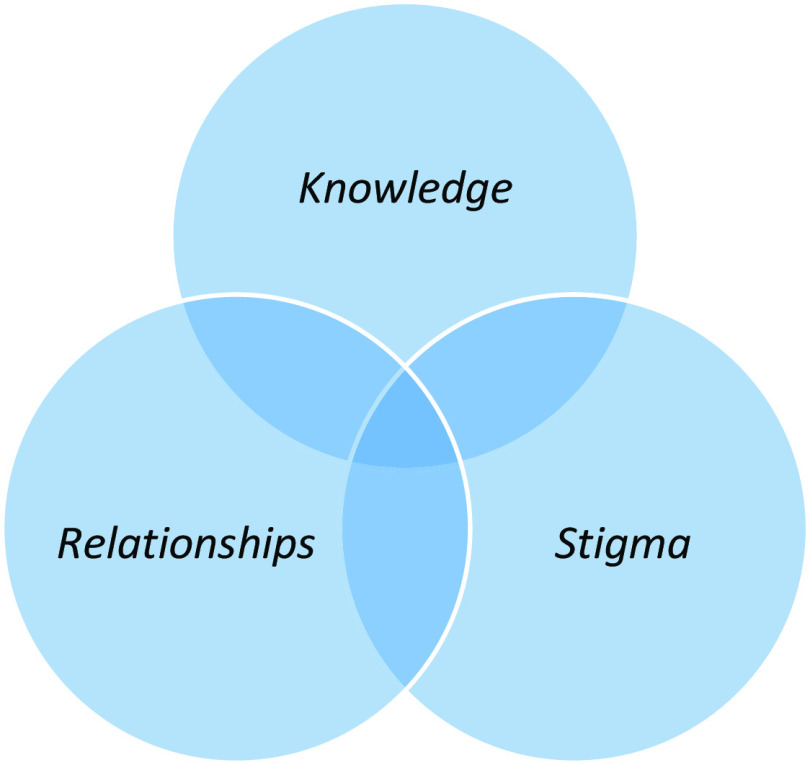
Intersectional knowledge and beliefs about self and others.

The iterative process of thematic analysis, and discussion within the research team, highlighted links between the three themes - how *knowledge*, *stigma,* and *relationships* often intersect to facilitate or create barriers to accessing early intervention services. Interpreting the qualitative data across the primary studies yielded an overarching analytic theme of *intersectional knowledge and beliefs about self and others,* which represents the three overlapping themes and highlights the inherently interpersonal nature of stigma and relationships (see Figure 2).

Knowledge and likelihood of accessing further information are affected by stigmatized beliefs about psychosis, mental health care, and oneself as a person who may have psychosis and need to access services. Generalized and self-stigma beliefs are by definition dependent on dominant socio-cultural discourses (e.g., psychosis as shameful) as well as personal and professional relationships. These generalized and specific social relationships in turn influence the knowledge we access and privilege when making healthcare decisions. The intersectionality of *knowledge*, *stigma,* and *relationship* beliefs about self and others suggests that public health and healthcare initiatives that target these in combination are likely to be more effective than strategies that focus on any one area in isolation.

## Discussion

This is the first systematic review of the barriers and facilitators to accessing early intervention for psychosis services. A comprehensive search of the published and unpublished literature (with no date limits) yielded 10 papers, the majority of which were qualitative.

A recent review by O’Connell et al. [18] highlights factors likely to improve *implementation* of early intervention services. Our review complements this by identifying factors which influence whether people *seek access to* these services. Mental health stigma is a key barrier and predicts DUP. Structural service barriers then further delay access to specialist services, despite the introduction of access and waiting times standards [48]. A synthesis of the qualitative studies generated three themes which both hinder and facilitate access to services: *knowledge, stigma,* and *relationships*, and an overarching analytic theme of *intersectional knowledge and beliefs about self and others.*

These findings align with and extend the wider literature which suggests that limited knowledge about mental health delays access to services for people with psychosis [49, 50], and that mental health literacy alongside supportive social and professional relationships increases help-seeking, which may in turn reduce DUP and improve outcomes [51]. Like McGonagle et al. [52], we found that stigma plays a key role in whether people disclose early psychosis and seek access to services, and that this is affected by dominant socio-cultural expectations [53]. Our review suggests that public health and service level initiatives should target these factors in integrated approaches that acknowledge the links between knowledge, stigma, and relationships.

### Public health, service, and research implications

Mental health literacy campaigns (targeting *knowledge*) delivered in cultural context (to address culturally shaped *stigma*) and targeting local communities as a whole (to influence *social and professional relationships*) may be particularly effective. For example, healthcare in-reach to schools might strengthen the impact of accurate information about psychosis and treatment options by drawing on young people’s often strong and collective sense of social justice to challenge the shame that drives stigmatizing beliefs about psychosis [cf. 54], and engaging well-regarded people in the local community to speak about their experiences of psychosis and accessing services – parent, child, and clinician triads might be particularly compelling.

Targeted training on the early signs of psychosis, how to access information and services, and how to be interpersonally effective in these interactions, should be delivered to professional groups who may come into contact with young people experiencing early signs of psychosis. Given the barriers identified in the current study, this should include primary care clinicians, emergency services, and education/immigration officials working with international students.

Secondary care services are likely to be more effective when clinicians are able to prioritize the development of supportive and trusting relationships with young people, shared decision-making, and flexible service delivery. These are of course built into service models for early intervention services, but are at risk when caseloads increase beyond recommended levels. The growing inclusion of peer support workers and befriending schemes in these teams is particularly welcome given the likely impact on knowledge, stigma, and relationships [55–57]. Routine clinical practice within these services should be extended to include culturally sensitive exploration of self-stigmatizing beliefs, and modeling of alternative ways of understanding and responding to psychosis, as a means of securing tentative engagement with young people.

In terms of research, we now need longitudinal quantitative and qualitative studies of young people’s decision-making and behaviors from the first signs of at risk mental states, in order to examine the role of candidate individual, interpersonal and service-related factors that affect likelihood of seeking access to specialist services, and how these change and can be targeted over time.

## Conclusion

This review identifies key barriers and facilitators to *seeking access* to early intervention for psychosis services, and complements a recent review of the barriers and facilitators to *implementation* of these services [18]. Together, these reviews highlight public health, systemic, service and staff factors that may be targeted to facilitate access to early intervention services, with the aim of reducing DUP and improving outcomes for people with psychosis.

## Supporting information

Tiller et al. supplementary material Tiller et al. supplementary material

## Footnotes

1 Friskney et al. [19] examine barriers and facilitators to access for people from South Asian heritage specifically, and Loughlin et al. [20] review ongoing engagement once people have accessed early intervention services.

2 The second rater reviewed 10% of the initial 375 articles identified. Further studies were identified when the search was checked and updated following a helpful comment from a reviewer of this paper.

3 Thomas and Harden [40] report considerable variation in the reporting of qualitative syntheses in systematic reviews of qualitative studies, particularly in the extent of data and results presented. We follow these authors’ recommendation that all text recorded in the primary study “Results” be identified as data for potential coding.

4 Full coding manual is available on reasonable request.

## Supplementary material

The supplementary material for this article can be found at https://doi.org/10.1192/j.eurpsy.2023.2465.
