# Supplementary material for: Early intervention in psychosis services: A systematic review and narrative synthesis of barriers and facilitators to seeking access
Source: Eur Psychiatry. 2023 Nov 6;66(1):e92. doi: 10.1192/j.eurpsy.2023.2465 (PMC10755576; doi:10.1192/j.eurpsy.2023.2465)
Supplement: Tiller et al. supplementary material [file S0924933823024653sup001.docx]

**Supplementary material:** *Code book of* *descriptive themes and illustrative quotes (from primary qualitative studies)*

| Themes | Papers evidencing themes | Quotes from papers |
| --- | --- | --- |
| Knowledge | 1, 2, 3, 4, 5, 6, 7 | *6. “I thought it was only a sad period, ‘just let things pass as they normally do’, until I’d gotten my head under control again.”*  *5. “I think I kept it to myself because I thought it was normal.”*  *1. “Others did not consider themselves as belonging to the TIPS target group mainly due to feeling insufﬁciently unwell (i.e. not sick enough) for TIPS.” (A)*  *1. “At times, they received treatment from GPs, psychologists, psychiatrists and school nurses for symptoms of anxiety and depression, but health-care professionals failed to correctly detect and diagnose psychosis.”* (A)  *3. “Joseph emphasised his open-mindedness to multiple explanations of his experiences… However, it is possible that Joseph was not entirely satisfied with the range of explanations provided, since he described seeking sources of additional information in his search for meaning.” (A)*  *3. “Joseph explained how the support provided by the EIP service and his personal research had facilitated self-reflection and knowledge.” (A)* |
| Stigma | 1, 2, 3, 4, 5, 6, 7 | *5. “Six participants discussed that shame and fear of stigma in relation to mental illness and how this made them less inclined to disclose symptoms and seek appropriate treatment.” (A)*  *6. “What I was most nervous about . . . to be labelled as crazy, afraid of being locked up, and not being let out again. It took a lot of courage to tell my general practitioner.”*  7*. “She presented with a long history of untreated psychosis and reported not seeking help earlier as mental illness was considered a taboo back home.” (A)*  *6. “I still ﬁnd it difﬁcult to accept that I have it, more because I’m worried that when I tell people about it, they label me crazy.”*  *5. “… so you’re just kind of afraid of being stigmatised by other people … you just know there are prejudices about all these things.”*  *3. “Frank talked about accessing the EIP service in a way that suggested this posed a threat to his previous sense of self, one strongly associated with dominant narratives of masculinity and the non-expression of emotion.” (A)*  4. “*I think for Asian people it’s quite difficult having a mental health problem . . . Asian people aren’t as accepting if you have a mental health problem and treat you very differently.”* |
| Relationships | 2, 3, 4, 5, 6, 7 | *3. “…facing numerous barriers to receiving support as she was dismissed by health care professionals and her family.” (A)*  3*. [216-219] “But the more time that they don’t give up on you (1) if that makes any sense, the more times that they stay there and when you do your stupidness they kind of don’t react to it, it kind of makes you realise that you do need a service.”*  *2.* ***“****Even when I haven’t come for like months on end, they always end up taking me back, when I feel like they’re going to think I’m using them but they’re always like, ‘This is what we’re here for’, so I really appreciate that. And they’re –they’re good, they’re ﬂexible and they’re understanding.”*  *3... “I told my family members (1) my sister but they, they didn’t believe me at first, they said it might be nothing.”*  *5. “Parents also assisted in ﬁnding, motivating for and visiting appropriate services.” (A)*  *7. “Despite reporting loneliness, she had one supportive friend in Montreal (also from her country) whom she considered to be like a sister. This friend occasionally accompanied her to appointments. Ms E was involved in a church that offered her emotional and instrumental (e.g. clothing) support.” (A)* |

*Note.* 1=Bay et al. (2016); 2=Cowan et al. (2020); 3=Harris (2016); 4=Islam et al. (2015); 5=Jansen et al. (2015); 6=Jansen et al. (2018); 7=Lee et al. (2016); (A)=Author quotes
